# Heights

**Published:** 1877-02

**Authors:** 


### HEIGHTS.

| | Feet. |
|---|---|
| St. Antoine Column, Rome | 135 |
| Trajan's Column, Rome | 145 |
| Smithsonian Institute, Washington City | 145 |
| Napoleon Column, Paris | 150 |
| Washington Monument, Baltimore | 180 |
| The Great Obelisk, Thebes | 200 |
| Bunker Hill Monument, Boston | 220 |
| Column of Delhi | 262 |
| Trinity Steeple, New York | 264 |
| New Dome of the Capitol, Washington | 300 |
| Dome of St. Paul's, London | 320 |
| The "Adriatic" Steamship, stood on end | 354 |
| Tower of Madonna, Rome | 465 |
| Great Pyramid, Egypt | 480 |
| National Monument, Washington | 517 |
| The "Great Eastern" stood on end | 684 |

And taller still is an honest man in a "panic;" but towering out of sight, and above all these, is that man, of low degree in life, who sees his children, day by day, pining away for want of necessary food, when the wages of sin would make them rich in any hour.

